# Can Health Human Capital Help the Sub-Saharan Africa Out of the Poverty Trap? An ARDL Model Approach

**DOI:** 10.3389/fpubh.2021.697826

**Published:** 2021-06-10

**Authors:** Qiu-Su Wang, Yu-Fei Hua, Ran Tao, Nicoleta-Claudia Moldovan

**Affiliations:** ^1^School of Economics, Qingdao University, Qingdao, China; ^2^Qingdao Municipal Center for Disease Control & Preventation, Qingdao, China; ^3^Finance Department, West University of Timisoara, Timisoara, Romania

**Keywords:** health human capital, poverty trap, Sub-Saharan Africa, ARDL, threshold

## Abstract

This article explores the impact of health human capital on the poverty trap in Sub-Saharan Africa by autoregressive distribution lag model. In the long run, there is no evidence that health human capital can help the Sahara out of the poverty trap. While health human capital has a significant effect on poverty reduction in the short term. There is a threshold effect in the poverty reduction model of healthy human capital. When the economic development level reaches the threshold, the effect of poverty reduction is more obvious and deeper. The extended Solow economic growth model also proved that if the external human capital breaks through the threshold, it can make developing countries get rid of the poverty trap. Therefore, the economic development brought about by health care expenditure must benefit the poor in Sub-Saharan Africa and allow them to enjoy the welfare of social security.

## Introduction

The COVID-19 (coronavirus disease 2019) has caused enormous cost to mankind, reversing the hard-won results of eliminating global poverty. According to the Poverty and Common Prosperity report, the poverty rate is expected to rise in 2020, which is measured by the international poverty line. There are 88 million to 115 million people likely to fall back into extreme poverty as a result of the COVID-19 epidemic, which is the first phenomenon since 1998 and ending more than 20 years of continuous progress. The projected poverty rate in 2020 is between 9.1 and 9.4%, similar to that in 2017. Therefore, the impact of COVID-19 is expected to reverse the progress of eliminating extreme poverty for at least 3 years.

In Sub-Saharan Africa, poverty reduction is slower than the global average, but some countries have also made impressive progress. For example, during 2004–2016, the extreme poverty rate in Ethiopia decreased by 7%, confirming a positive trend since the early 2000s. In Kenya, the proportion of people living below the international poverty line dropped from 44% in 2005 to 37% in 2015. And it fell from 23 to 13% between 2009 and 2015 in Namibia. Even so, among the world economies that can measure poverty, 18 of the 20 poorest countries are still in Sub-Saharan Africa, where extreme poverty is increasingly concentrated. Before the COVID-19 was prevalent, millions of people in Sub-Saharan Africa were reluctantly able to escape extreme poverty. People who have just emerged from extreme poverty are very vulnerable to epidemics, conflicts, and climate change and are very easy to turn back poverty. Apart from South Asia, Sub-Saharan Africa is the second most severely affected area by COVID-19. It is estimated that 26 million to 40 million people will be plunged into extreme poverty, which will be 7 years slower to achieve the goal of zero poverty by 2030.

There are still some more serious health problems in Sub-Saharan Africa. The incidence rate of malaria in this region is 219.13%, accounting for 90% of the total number of malaria deaths worldwide. More than 60% of the world's human immunodeficiency virus/acquired immune deficiency syndrome (HIV/AIDS)–infected people are in Sub-Saharan Africa, and women and girls are still the most affected groups. Compared with the global level, Sub-Saharan Africa has the highest mortality rates for children younger than 5 years and infants. In contrast, health care in Sub-Saharan Africa is inadequate. In 2018, Sub-Saharan Africa's health expenditure [% of gross domestic product (GDP)] accounted for 5.09%, which is far below 12.46% of the Organization for Economic Cooperation and Development (OECD) countries. Funding for the development of the health care system has been reduced, which leads to inadequate infrastructure maintenance and the shortage of personal protective equipment, ventilators, intensive care unit (ICU) beds and other medical necessities (1). Pheeha and Umakrishnan (2) discovered that South African residents' access to high-quality health care is a privilege, which is open to only a few people.

The WHO supports the view that improving the health and longevity of the poor is a goal, which is a fundamental goal of economic development and also a target to achieve the goal of poverty reduction. Health is an essential factor of human capital, which can have a positive impact on economic growth (3, 4). A perfect medical social security system will increase a country's health human capital. From a macro point of view, health investment can increase government health investment and control environmental pollution, which is used to achieve the purpose of reduce mortality, improve fertility, and prolong life span. At the micro level, health investment can improve the physical condition of workers and optimize their consumption decisions by increasing food and medical expenses. Health human capital investment can create a living environment through social security measures and achieve inclusive growth for the poor, which may reverse the increase in poverty caused by the COVID-19 crisis and prevent other vulnerable groups from falling into extreme poverty. Therefore, in addition to continuing economic aid, it is also a good way to provide health human capital assistance to help the Sahara region get rid of poverty trap.

The study makes three contributions. First, Sub-Saharan Africa has always been the region with the most serious poverty problem in the world, and the impact of COVID-19 is just like adding to one disaster after another, which brings great challenges to the local medical and health environment. Based on this, this article takes the government's health care expenditure as the investment of health human capital, and finds that it plays an active role in reducing the incidence of poverty. The conclusion encourages Sub-Saharan Africa to strengthen the health care system and prevent some vulnerable groups from falling into poverty trap by sudden COVID-19. Second, we observe the long- and short-term effects of health human capital on poverty by autoregressive distribution lag (ARDL) model. In the long run, health human capital does not show a positive effect. Current projections assume that Sub-Saharan Africa cannot achieve the sustainable development goal of reducing extreme poverty to <3%, unless income inequality is solved through more inclusive growth (5). In the short run, health human capital can significantly help Sub-Saharan areas out of the poverty trap. This is consistent with the extended Solow economic growth model. Finally, there is a threshold effect in the poverty reduction model, and the elasticity coefficient of health human capital is different before or after the threshold value. When the economic development level exceeds the threshold value, the impact of health human capital on reducing the incidence of poverty is deeper, and the effect is better.

The rest of this study is organized as follows: *Literature review* reviews the existing literature, whereas *Extended Solow growth theoretical model* presents the influence mechanism of human capital on poverty trap. *Methodology and data* describes the ARDL model in this article and the data description. *Empirical results* shows the findings of the study. *Conclusions* offers concluding remarks.

## Literature Review

Poverty is dynamic, which is increasingly regarded as a multilevel structure in recent years. The shocks of COVID-19 have deepened the poverty trap. Bich et al. (6) found that the poverty trap brought by COVID-19 is formed by the obstacles to prevention, the fragility of financial chaos, financial uncertainty, and the catastrophic cost that people are trying to cope with. Li et al. (7) studied the impact of COVID-19 on Rural China, especially in poor areas, and thought that it increased the vulnerability of farmers in poor areas to multidimensional poverty. According to Goenka and Liu (8), the endogenous epidemic of diseases determines the human capital accumulation, which is also one of the reasons for economic growth or poverty trap. Countries in poverty trap are mainly found in Sub-Saharan Africa, which have low human capital and high incidence rate of infectious diseases. This view also expounds the important role of human capital in poverty. Antonio et al. (9) concluded the existence of poverty trap and believed that it was determined by the level of per-capita capital stock.

Persistent poverty is related to poor health behaviors, and health is also a factor of human capital (10). Callander et al. (11) revealed that in the case of poor health or education, these capacity constraints may become obstacles to their labor force participation and lead to a decline in the quality of life. Daepp and Arcaya (12) also proved that poor health limits an individual's ability to work, reduces economic opportunities, and leads to low income. Ogundari and Abdulai (3) showed that a better educated and healthier workforce is more likely to create and adopt new technologies. Oshio (13) searched for a poverty line that maximized the likelihood of the logistic regression model to explain poverty. The results suggest that there is a risk that the conventionally defined poverty line may underestimate poverty in terms of population health. Patel et al. (14) found that economically disadvantaged people were more likely to live in overcrowded homes, which is a risk factor for lower respiratory tract infections.

The emphasis on health and medical care of one country is also the main reason to reduce poverty. Dirgha and Sabina (15) supported the role of public health expenditure in reducing rural poverty. Caminada (16) still proved a strong negative relationship between the level of social expenditure and poverty. Gnangnon (17) pointed out that fiscal redistribution has a positive impact on poverty in developing countries, but for the least developed countries, it leads to the reduction of poverty rate. Viju and Wullianallur (18) showed that through careful investment in health care, American states can increase income, GDP, and productivity and reduce poverty.

In the economic growth model, health and material human capital have an impact on economic growth (19–22), and economic growth is an important means of poverty reduction (23–27). Therefore, the impact of health human capital on poverty through the regulation of economic growth has also been widely concerned. Because economic growth reduces poverty, Anand and Ravallion (28) considered the positive impact of GDP growth on health. Therefore, for poorer countries, the relationship between GDP growth and public health should be stronger, if the government takes appropriate measures to increase health expenditure and technological progress, while reducing poverty rate, infant mortality rate, and fertility rate. Jayadevan (29) revealed that health human capital in developing countries can have a significantly positive impact on economic growth. Cooray (30) proved that health has a significantly positive impact on the economic growth of middle- and high-income countries. At the same time, the health capital of low-income economies can obtain a significant position only through the interaction of education and health expenditure. Biggs et al. (31) revealed that GDP growth has a significantly positive impact on population health. However, the strength of this relationship is strongly affected by changing levels of poverty and inequality. Akingba et al. (32) concluded that if health capital expenditure is increased, Singapore's economic growth can be significantly improved. This can ultimately have a significant impact on human productivity, thereby increasing per-capita output. If there are feasible strategies to reduce diseases in low-income groups, such as monitoring the health status of the poor and extending Medicaid, Kim (33) suggested that this can make the poor get better medical services.

In addition, there is a threshold effect in the relationship between human capital and economic growth. As health investment may crowd out physical capital investment and affect the accumulation of physical capital, Gong et al. (34) found that excessive investment in health may have a negative impact on economic growth. Çepni et al. (35) proved that the effect of human capital on economic growth is positive when the ratio of human capital to material capital is low, whereas if it exceeds a threshold, the effect becomes negative. Zhang et al. (36) also pointed out the existence of this threshold effect. Whether the level of human capital is too low or too high, social security is conducive to sustained economic growth. However, if the level of human capital is in the medium level, the role of social security is very weak. Bertrand (37) indicated that the economic benefits of the return on investment in education can be utilized in the region of the Economic Community of Central African States, which has not reached the level of human capital. Asongu et al. (38) suggested that policy makers can use policy thresholds to promote inclusive and sustainable development in Sub-Saharan Africa.

The prior literature provides some inspiration. First, although health is an element of human capital, whether it can benefit from the poor to achieve revenue growth is a problem worthy of discussion. Bekana and Keun (39) used the example of Ethiopia to confirm the point that pro-poor growth is good for poverty eradication if it can be achieved. Secondly, the existing research focuses only on the formation of poverty trap. However, poverty itself has a great continuity and is very vulnerable to its own influence. The ARDL–error correction model (ECM) model can analyze the long- and short-term effects of health on poverty and also observe the impact of poverty lag. Finally, the literature mainly studies the causal relationship between health and poverty, but the poverty trap theory also emphasizes the existence of threshold effect. Therefore, it is necessary to consider the threshold effect of economic growth in the poverty model.

## Extended Solow Growth Theoretical model

Consider an extended Solow growth model with health and education human capital. Assuming that the production function is

(1) $$\begin{array}{l} Y\left(t\right)=K{\left(t\right)}^{\alpha }E{\left(t\right)}^{\beta }H{\left(t\right)}^{\gamma }{\left(A\left(t\right)L\left(t\right)\right)}^{1-\alpha -\beta -\gamma } \end{array}$$

where *Y* is total output, *K* is total material capital, *E* is education human capital, *H* is health human capital, *L* is labor force, *A* is technical level, and *t* is time. α, β, and γ are positive parameters <1, and α + β + γ < 1. Suppose that the population *L* and technical level *A* of a country grow at a fixed growth rate *n* and *g*, respectively (40).

$ŷ,\hat{K},ê$ and $\overset{⌢}{h}$ are, respectively, used to express per-capita effective output, material capital, education human capital, and health human capital. According to Equation (1), Equation (2) can be obtained. Therefore, after controlling the material capital and educating human capital, there is a correlation between healthy human capital and economic output (41).

(2) $$\begin{array}{l} \overset{⌢}{y}\left(t\right)=\overset{⌢}{k}{\left(t\right)}^{\alpha }\overset{⌢}{e}{\left(t\right)}^{\beta }\overset{⌢}{h}{\left(t\right)}^{\gamma } \end{array}$$

**Figure 1:**
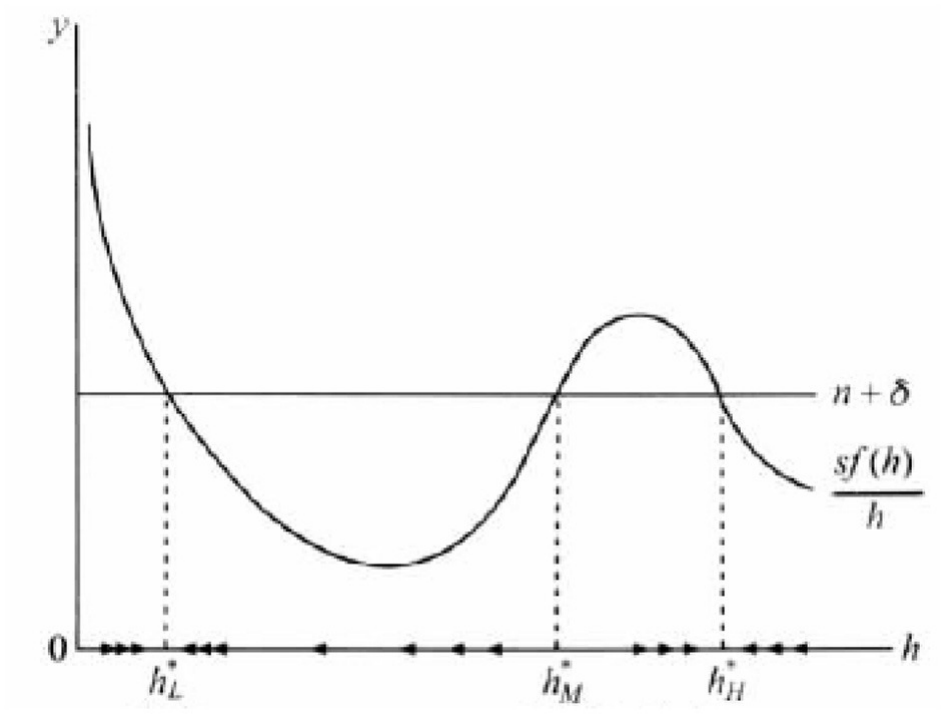
Poverty trap model of economic growth.

As we know, human capital in developing countries leads to a low level of stability, which results in the “poverty trap” of economic growth. If someone tries to break this stable state, the economy tends to return to a low level, it is called poverty trap. Figure 1 shows the poverty trap model of economic growth induced by insufficient human capital.

It is assumed that the production function *f(h)* presents diminishing returns when human capital *h* is very low, increasing returns for *h* in the middle region, and constant or decreasing returns when *h* is very high. Therefore, the average saving rate *sf(h)/h* has three intersections with the straight line (*n* + δ), respectively, delivered at h^*^L, h^*^M, and h^*^H, in which h^*^L is a stable state similar to the neoclassical model.

h^*^M is an unstable equilibrium state. As long as the initial human capital level *h* deviates from h^*^M, the deviation will be larger. The economy eventually tends to decrease in return, so the curve *sf(h)/h* and the linear (*n* + δ) transaction point h^*^H. Similar to h^*^L, h^*^H is a stable state. As long as the level of human capital is around h^*^H (*h* > h^*^M), it will converge to this point. h^*^L represents the low level of human capital. When *h* < h^*^M, economy will converge to the low level of stable state h^*^L, which leads to the economy developing slowly. If there is an influx of foreign human capital, as long as it is not enough to break through the critical value of h^*^M, it will return to h^*^L in the long run, which is the so-called poverty trap. If the growth model of poverty trap is in line with the reality, developing countries must make the level of human capital exceed the critical value h^*^M to get rid of the poverty trap. Therefore, only when the government increases the investment of health human capital can the Sahara countries break through the economic shackles and get rid of the poverty trap.

## Methodology and Data

### Methodology

Poverty itself has a lot of stickiness, and it is easy to be affected by the early stage, so it will take a long time for human beings to reduce poverty. The impact of health human capital on poverty must be considered separately in the short or long term, and the ARDL boundary test method proposed by Pesaran et al. (42) totally matches our idea. Compared with the traditional test method, the ARDL method has the following advantages. First, the sequence to be tested does not need to be of the same order, which can be I (0) or I (1). Second, ARDL model is robust enough to test small samples, and when the explanatory variables are endogenous variables, it can also get unbiased and effective estimates. Third, the ARDL model can derive the dynamic ECM through simple linear transformation and combine short-term with long-term effect (43).

The first step is to test the stationarity of time series. If the sequence is I (0) or I (1) process, the ARDL boundary cointegration test can be carried out by Equation (3) (44).

(3) $$\begin{array}{l} \Delta LnP_{t}=\alpha_{0}+\overset{n}{\underset{i=1}{\sum }}\alpha_{1i}\Delta LnP_{t-i}+\overset{n}{\underset{i=0}{\sum }}\alpha_{2i}\Delta LnH_{t-i}\\ \;+\overset{n}{\underset{i=0}{\sum }}\alpha_{ki}\text{Contro}{\text{l}}_{t-i}+\alpha_{6}\Delta LnP_{t-1}\\ \;+\alpha_{7}\Delta LnH_{t-1}+\alpha_{m}\Delta \text{Contro}{\text{l}}_{t-1}+\mu_{i} \end{array}$$

*P* is the incidence of poverty, *H* is the health human capital, control is the control variables, and Δ is the first-order difference item; α_1_, α_2_, α_*ki*_ is the short-term dynamic relationship; and α_6_, α_7_, α_*m*_ is the cointegration relationship between variables.

If it is confirmed that there is cointegration relationship between variables, the second step is to analyze the long-term relationship between variables through ARDL model. It is worth noting that the coefficient estimation in Equation (3) is only used to determine whether there is a long-term relationship, and the coefficient in Equation (4) is used to estimate the size of long-term effect.

(4) $$\begin{array}{l} \Delta LnP_{t}=\alpha_{0}+\overset{n}{\underset{i=1}{\sum }}\alpha_{1i}LnP_{t-i}+\overset{n}{\underset{i=0}{\sum }}\alpha_{2i}LnH_{t-i}\\ \;+\overset{n}{\underset{i=0}{\sum }}\alpha_{mi}\text{Contro}{\text{l}}_{t-i}+\mu_{i} \end{array}$$

The poverty trap is explained by the lagged value of poverty itself and the current or lagged value of explanatory variables. α_1*i*_ is the influence of poverty's lag value on its current period. α_2*i*_ is the long-term effect of the change of health human capital on poverty. α_*mi*_ is the long-term effects of changes in control variables on poverty.

The third step is to derive ARDL-ECM model, and the Equation (5) is to analyze the short-term effects between variables.

(5) $$\begin{array}{l} \Delta LnP_{t}=\alpha_{0}+\overset{n}{\underset{i=1}{\sum }}\alpha_{1i}\Delta LnP_{t-i}+\overset{n}{\underset{i=0}{\sum }}\alpha_{2i}\Delta LnH_{t-i}\\ \;+\overset{n}{\underset{i=1}{\sum }}\alpha_{ki}\text{Contro}{\text{l}}_{t-i}+ECM_{t-1}+\mu_{i} \end{array}$$

The coefficients are the short-term effects of the variables on poverty. ECM_*t*−1_ is the lag error correction factor, which indicates the self-correction speed of the economic system.

### Data

This article is based on the data of Sub-Saharan African countries for 2000–2019, and it is retrieved from world development indicators. The choice of starting period is limited by data availability. According to the latest poverty line standards published by the World Bank, the standard line of extreme poverty is <$1.9 per person per day; the poverty line of developing countries is <$3.2, and that of the developed countries is <$5.5. Sub-Saharan African countries are all developing countries. Therefore, this article calculates the proportion of the poor population (P) by using the poverty line below $3.2 per person per day. Health human capital (H) is measured by domestic general government health expenditure (% of GDP). Health expenditure is a major determinant of health outcomes (45, 46).

**Figure 2:**
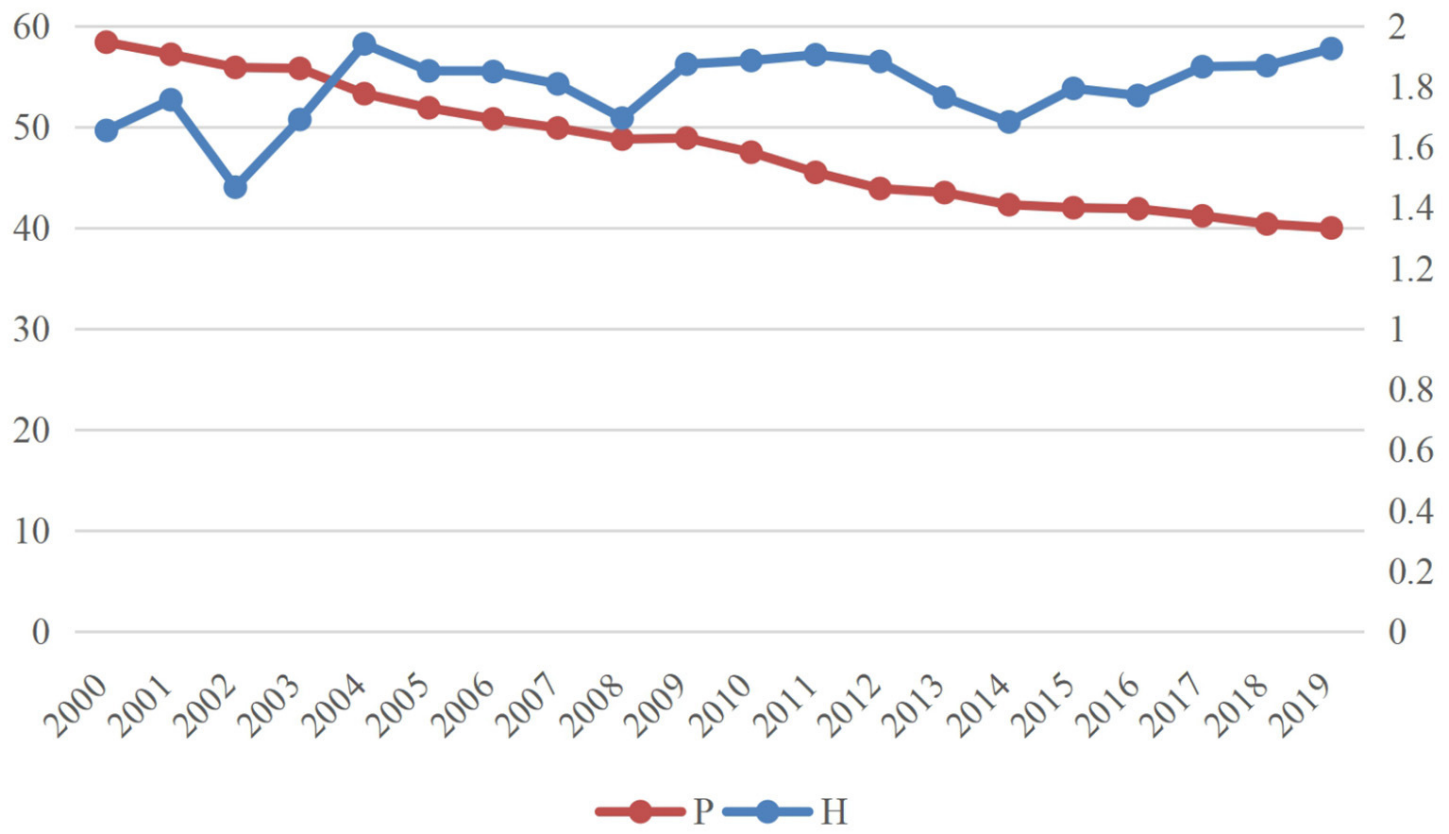
Trends in health human capital and poverty rates from 2000 to 2019.

Figure 2 shows trends in health human capital and poverty rates in Sub-Saharan Africa from 2000 to 2019. It can be seen that the incidence of poverty in Sub-Saharan Africa is declining, from 58.4% in 2000 to 40% in 2019. Sub-Saharan Africa has benefited from the efforts of the international community and made effective progress in poverty reduction. Based on historical links and practical needs, developed countries provide aid to Africa through multilateral institutions (such as the World Bank, the European Union, the African Development Bank, the International Monetary Fund, and the United Nations) and bilateral assistance. The 35 members of the OECD Development Assistance Committee are the main donors of developed economies to Africa. Therefore, with the joint efforts of African countries and the international community, their economic development achievements cannot be ignored.

The health human capital displays a low fluctuation, only rising from 1.65% in 2000 to 1.92% in 2019, while the current health expenditure (% of GDP) of middle- to high-income countries is 5.74%, which is three times that of Sub-Saharan Africa. As a result, the health care expenditure in Sub-Saharan Africa is very poor, which inevitably leads to the low level of health human capital (47). Investment and recurrent expenditure in the health sector are affected by funding shortages, high costs, limited government revenue, and poverty. As of 2019, the funding gap for health care sector in Sub-Saharan Africa is US $66 billion. Meanwhile, poor sanitation led to Sub-Saharan Africa's burden of global incidence rate (tuberculosis, malaria, and HIV/AIDS), mortality (maternal and child mortality), and low life expectancy.

In addition, we have control of variables. Since the beginning of the 21st century, population aging has put forward higher requirements for the improvement of social security system in developing countries, and the aging problem in Sub-Saharan Africa is becoming more and more serious (48). For example, the average age of farmers is estimated at 60 years in Kenya. This is one of the main reasons for falling into the poverty trap. In this article, the population 65 years or older (% of the total population) is regarded as the standard of aging. Education and health are considered to be the two major investments in the concept of human capital. Education (EDU) also has an impact on a country's economic development (49). The role of education expenditure (% of GNI) in poverty reduction is also crucial (50). Obviously, the GDP is also an important indicator of a country's poverty situation (25, 26). Table 1 shows the descriptive statistics of variable. The average incidence of poverty is 72.7105%, which is a very high proportion and deserves attention. The standard deviations for *P* and GDP show that they change greatly over time. The positive skewness of the values indicates that *P* and AGE have right-skewed distributions. The kurtosis of all variables is >1.5, which reveals that the distributions are far from normal. Furthermore, the Jarque–Bera index indicates that variables are significantly non–normally distributed at the 1% level. The empirical analysis uses the natural logarithm of the variables.

**Table 1:** Descriptive statistics for variables.

**Variables**	**Mean**	**Min**	**Max**	**Std**.	**Skewness**	**Kurtosis**	**Jarque-Bera**
Poverty rate (*P*)	72.711	66.700	79.100	4.176	0.122	1.628	1.538^***^
Health human capital (*H*)	1.790	1.468	1.941	0.114	−1.167	4.284	5.615^***^
*GDP*	1511.438	1220.836	1698.456	165.463	−0.497	1.799	1.924^***^
Education expenditure (*EDU*)	3.373	3.074	3.611	0.154	−0.213	1.978	0.971^***^
Aging of population (*AGE*)	2.947	2.905	3.005	0.032	0.637	2.003	2.074^***^

*** *Represent the significance levels of 1%*.

## Empirical Results

### ARDL Empirical Results

Although ARDL model does not require all sequences to be single integral of the same order, it requires that the order of variables cannot exceed I (1). Pesaran et al. (44) proposed that the boundary value of *F* statistic is calculated based on the sequence I (0) or I (1). If the sequence exceeds I (1), the *F* statistic to determine whether there is a cointegration relationship will be invalid. In this article, the augmented dickey-fuller (ADF) test is used to determine the order of single integer (51). The results are shown in Table 2, although each sequence is not a single integration process of the same order, and the highest order is less than I (1), which satisfies the precondition of ARDL boundary cointegration test.

**Table 2:** ADF Unit root test.

**Variables**	**t-statistics**	***p*-value**	**Conclusion**
*P*	0.285	0.909	unstable
Δ(*P*)	−3.830	0.012	stable
*H*	−2.947	0.059	stable
Δ(*H*)	−5.361	0.001	stable
*GDP*	1.082	0.999	unstable
Δ(*GDP*)	−3.929	0.003	stable
*EDU*	−3.682	0.015	stable
Δ(*EDU*)	−3.271	0.036	stable
*AGE*	−0.117	0.988	unstable
Δ(*AGE*)	−15.721	0.000	stable

Δ*Represents the first order lag of the variable*.

The ADF test results showed that *H* and EDU were I (0), and other variables were I (1). The traditional Engel and Granger and Johansen cointegration test does not work. For this reason, this article uses the boundary cointegration test method proposed by Pesaran et al. (42) for further analysis. AIC, SC information criterion, and bounds test provide an effective method to determine the lag order of ARDL-ECM model. The optimal lag order of each difference item is determined to be 2. The *F* statistic in the bounds test method can determine the existence of cointegration relationship, and the results are shown in Table 3.

**Table 3:** Co-integration test.

**Significance level**	**10%**		**5%**		**1%**	
	**I (0)**	**I (1)**	**I (0)**	**I (1)**	**I (0)**	**I (1)**
critical value	2.450	3.520	2.860	4.010	3.740	5.060
F-statistic	10.408					

The *F* statistics of boundary test are higher than the highest critical value of 1% level, indicating the long-term cointegration relationship between poverty and explanatory variable. Therefore, the long- and short-term coefficients among variables can be further estimated by using the ARDL model.

Table 4 shows the long-term estimation results of ARDL (1,1,2,2). The elasticity coefficient of *H* is −0.008, which is not statistically significant. In the long run, health human capital cannot help the Sahara out of the poverty trap. Gong et al. (34) proved that the introduction of health into Romer (1986) endogenous growth model may lead to the existence of development trap, which means that only in economies with initial capital higher than a certain level growth will continue. Most countries in Sub-Saharan Africa are at a low level of economic development, which has inhibited the effectiveness of governments in improving income and inequality in poverty reduction (52). Therefore, the introduction of health human capital into the economy may lead to poverty trap in the economy. Fan et al. (50) also put forward that government expenditure on health has no significant impact on agricultural productivity growth or rural poverty reduction. It is not simply true that public health spending is unimportant, but government effectiveness and bureaucracy quality determine whether public spending will have a major impact on health outcomes (53). Akinlo and Sulola (54) suggested that health spending has not translated into an improvement in the mortality rate of children younger than 5 years in Sub-Saharan Africa, which may reflect the high level of corruption and alternatives to public health spending. In a nutshell, health human capital cannot help Sub-Saharan Africa out of poverty trap in the long run, which is closely related to local income inequality, government corruption, and inefficiency.

**Table 4:** Long-term estimation results of ARDL.

**Variables**	**Coefficient**	**Std**.	**t-statistics**	***p*-value**
Δ(*H*)	−0.008	0.022	−0.392	0.702
Δ(*GDP*)	−0.436	0.146	−2.996	0.010
Δ(*EDU*)	−0.049	0.042	−0.951	0.359
Δ(*AGE*)	0.980	0.450	2.176	0.049
*C*	−0.002	0.003	−0.828	0.423
*R^2^*	0.428			

Δ*Represents the first order lag of the variable*.

As expected, economic growth and slowing down the aging have had a significantly positive impact on getting rid of the poverty trap; elasticity coefficients of GDP and AGE are −0.4359 and 0.9801, respectively. Facts have proved that economy and young labor force are the important factors to get rid of poverty. The elasticity coefficient of EDU is negative, but it is not statistically significant, which has no return on poverty reduction for the time being.

Table 5 shows the short-term estimation results of ARDL (1, 1, 2, 2). The elasticity coefficient of the *P* (−1) is 0.851, which is significant at the level of 10%. It verifies that the inducement of poverty trap is easily affected by the previous period and has considerable stickiness. The medical poverty trap refers to a state of self-continuation, in which the poor face greater risk of ill health. In turn, poor health increases the likelihood of poverty through out-of-pocket costs for public and private health services (55, 56). The road to get rid of the poverty trap in Sub-Saharan Africa is very difficult.

**Table 5:** Short-term estimation results of ARDL.

**Variables**	**Coefficient**	**Std**.	**t-statistics**	***p*-value**
Δ [*P* (−1)]	0.851	0.199	4.271	0.051
Δ(*H*)	0.014	0.009	1.585	0.254
Δ [*H* (−1)]	−0.038	0.010	−3.868	0.061
Δ(*GDP*)	−0.422	0.051	−8.264	0.014
Δ [*GDP* (−1)]	0.623	0.123	5.066	0.037
Δ [*GDP* (−2)]	0.170	0.059	2.885	0.102
Δ(*EDU*)	0.049	0.017	2.861	0.104
Δ [*EDU* (−1)]	0.065	0.022	2.871	0.104
Δ [*EDU* (−2)]	0.045	0.018	2.493	0.130
Δ(*AGE*)	−1.713	0.171	−10.042	0.010
Δ [*AGE* (−1)]	1.462	0.265	5.516	0.031
Δ [*AGE* (−2)]	1.156	0.268	4.307	0.050
*ECM* (−1)	−2.669	0.274	−9.754	0.010
*C*	−0.007	0.002	−4.107	0.055
*R^2^*	0.993			

Δ*Represents the first order lag of the variable*.

The elasticity coefficient of the *H* (−1) is −0.038, which is significant at the level of 10%, indicating that health investment is of great help to get rid of the poverty trap. In order to treat sick family members, nearly one-third of households had to use savings or loans to pay for medical expenses in a specific group of low-income countries (11). In Sub-Saharan Africa, government health spending focuses on the prevention and treatment of HIV/AIDS, malaria-related diseases, and advocacy for a healthy living environment. It has a significant impact on long-term growth and poverty reduction and has a direct impact on the well-being of the poor. The Ugandan government has substantially increased its budget allocation for primary health care through the poverty alleviation action fund, which is dedicated to improve the welfare of the poor. After the outbreak of Ebola in West Africa in 2014–2015, the World Bank helped establish the African Center for Disease Control and many national public health institutions, which is now playing a role in dealing with COVID-19. Strengthening the primary health care system can reduce disease-related financial risks and improve equity, thereby helping countries provide quality and affordable health services, which has a great effect on getting rid of the poverty trap.

Among the control variables, GDP has a negative impact on the incidence of poverty in the current period, and the elasticity coefficient is −0.422, which is significant at the level of 10%. In recent years, inclusive growth for the poor may have great benefits in reversing the aggravation of poverty caused by diseases (57) and preventing other vulnerable groups from falling into extreme poverty. Edriss and Chiunda (58) found that the poverty level in Malawi seems to have a significant response to GDP. In the long run, economic growth is the key to alleviating extreme poverty, because it creates resources to increase income.

The elasticity coefficient of EDU is positive, but not significant. The possible reason is that health care expenditure has a crowding-out effect on education. In the Sub-Saharan region, people are still in the stage of seeking to solve the problem of food and clothing, and they do not pay enough attention to education. The secondary school enrollment rate (% of the total population) only reaches 43% in 2019, compared with the world average of 76%.

The process of population aging hinders the effect of poverty reduction, the elastic coefficients of AGE (−1) and AGE (−2) are 1.462 and 1.156, respectively, which are significant at the level of 10%. No matter in the absolute sense, or relative to other groups, the elderly is relatively poor and prone to poverty. In Sub-Saharan African countries, a considerable number of elderly people continue to engage in economic activities and are active in agriculture. Capacity constraints make their economic output very low (49). Callander and Schofield (59) proved that for those younger than 70 years, developing heart disease is associated with an increased risk of falling into both income poverty and multidimensional poverty. If we want to reduce poverty and hunger sustainably in the region, population aging is a serious problem.

According to the poverty trap theory, there is a threshold effect in all countries, regions, and individuals. Whether it is production technology, material capital, human capital, or other medical and social assistance, economic mechanism will work only after this threshold is reached, and people can get rid of poverty trap. The specific threshold value of different countries, regions, or individuals depends on the specific politics, economy, culture, environment, and resources. Therefore, this article takes GDP as the threshold value, and the results are shown in Table 6.

**Table 6:** Threshold test results.

**Threshold value**	**Variable**	**Coefficient**	**t-statistics**	***p*-value**
*GDP* < 7.3843	*H*	−0.764	−2.284	0.039
*GDP*≥7.3843	*H*	−1.295	−3.890	0.002
	*AGE*	2.051	1.062	0.306
	*EDU*	1.379	3.161	0.007
	*C*	−0.337	−0.171	0.867
	*R^2^*	0.911		

**Figure 3:**
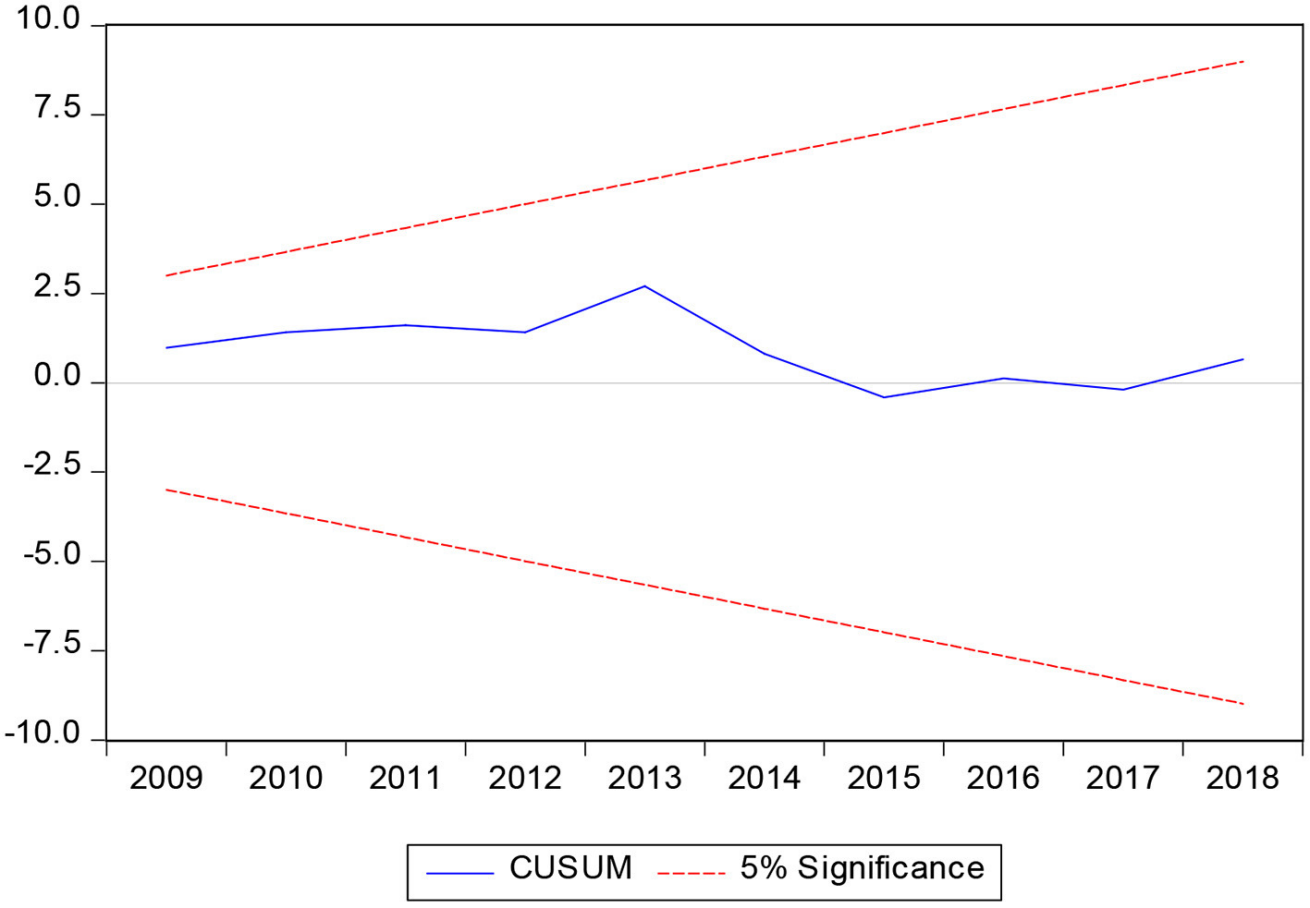
CUSUM stationarity test.

**Figure 4:**
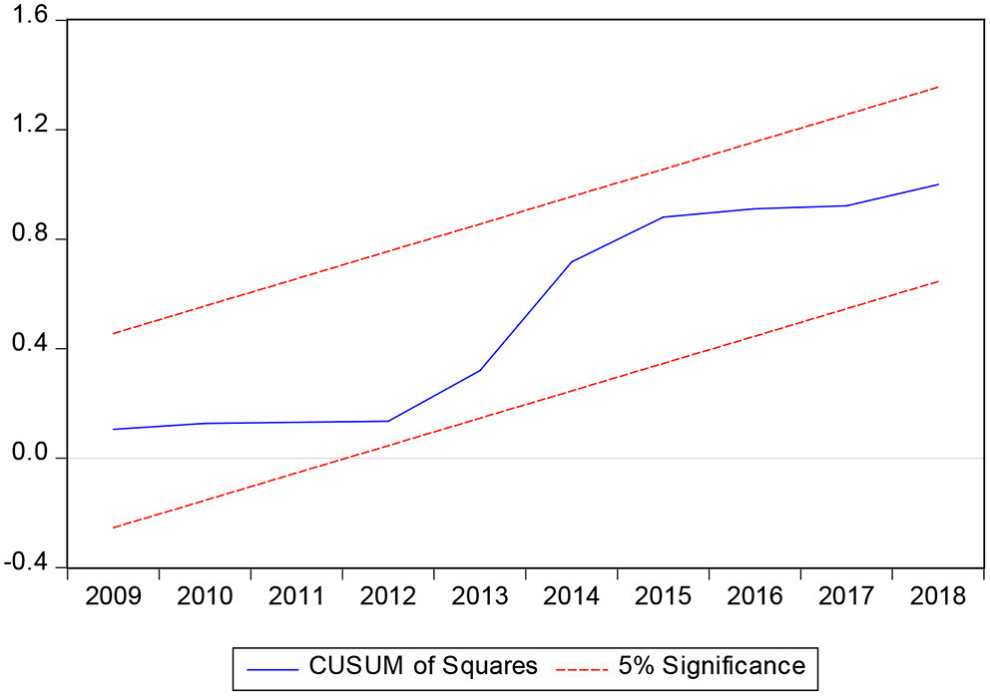
CUSUMSQ stationarity test.

In the process of establishing long-term equilibrium equation and cointegration test, the parameters to be estimated are usually fixed by default. In fact, the time-series model may have the problem that the estimated parameters fluctuate with time. In order to avoid that the reliability of the model setting is questioned due to the instability of parameters, this article tests the stability of the parameters by CUSUM and CUSUMSQ. These two tests are conducted at the 5% significance level. From Figures 3, 4, it can be seen that CUSUM and CUSUMSQ statistics are not deviated from the boundary range, which shows that the coefficients of the ARDL model are stable and the reliability is high.

The threshold test results show that when GDP <7.3843, the elasticity coefficient of *H* is −0.764; When GDP ≥7.384, the elasticity coefficient of *H* is −1.295, which are all significant at the level of 10%. That means the poverty reduction effect of health human capital is affected by the level of economic development, and there is a threshold effect. The effect of poverty reduction is related to the level of economic development. Because of the existence of threshold effect, the economy has multiple equilibriums. Countries that cannot make full use of health human capital will fall into the poverty trap, whereas abundant capital will get more wealth and jump out of the poverty trap. Globally, low-income levels inhibit the effectiveness of improving income and inequality in poverty reduction in many African countries (52).

### Robustness Check

Poverty gap reflects the degree of income inequality in Sub-Saharan Africa, which refers to the ratio of the average income below the official poverty line. By 2017, the poverty gap in Sub-Saharan countries was 15.6%, whereas the world average was 2.9%. If Sub-Saharan countries want to get rid of the poverty trap, narrowing the poverty gap should also be considered. Therefore, robustness check takes poverty gap as dependent variable in Table 7 and provides a new idea for poverty reduction programs in Sub-Saharan countries.

**Table 7:** ARDL test results (poverty gap as dependent variable).

	**Variable**	**Coefficient**	**Std**.	**t-statistics**	***p*-value**
Long-term estimates	Δ(*H*)	0.009	0.059	0.159	0.876
	Δ(*GDP*)	−1.838	0.184	−9.982	0.000
	Δ(*EDU*)	−0.251	0.109	−2.309	0.037
	Δ(*AGE*)	−2.890	0.929	−3.110	0.008
	*C*	−0.008	0.008	−0.988	0.341
	*R2*	0.579
Short-term estimates	Δ [*PG* (−1)]	−0.108	0.260	−0.416	0.699
	Δ(*H*)	−0.160	0.122	−1.315	0.259
	Δ [*H* (−1)]	−0.067	0.070	−2.951	0.096
	Δ(*GDP*)	−0.623	0.527	−1.183	0.302
	Δ [*GDP* (−1)]	−0.208	0.644	−0.324	0.762
	Δ [*GDP* (−2)]	0.618	0.361	1.711	0.162
	Δ(*EDU*)	0.256	0.229	1.121	0.325
	Δ(*AGE*)	−1.934	1.059	−1.826	0.142
	Δ [*AGE* (−1)]	0.624	1.056	0.591	0.587
	Δ [*AGE* (−2)]	2.448	1.723	1.421	0.229
	*ECM* (−1)	−1.104	0.421	−2.626	0.058
	*C*	−0.030	0.017	−1.764	0.153
	*R^2^*	0.908			

Δ*Represents the first order lag of the variable*.

In the long run, health human capital has not played a significant role in improving the income gap in Sahara countries. However, in the short term, the elasticity coefficient of *H* (−1) is −0.067, which is significantly negative at the level of 10%. It is similar to the conclusion with Table 5, which proves the effectiveness of the results. Because of the widespread inequality in health, education, and living conditions, Africans have suffered more than others in terms of human development. In recent years, many Sub-Saharan African countries have experienced rapid economic growth, which raises sharp questions about the distribution of opportunities and benefits (49). The result is an imbalance in poverty reduction among different groups, such as a big gap between urban and rural areas. In 1992, almost 60% of the rural population lived below the poverty line, whereas only 28% of the urban population did. In 2018, nearly 40% of the rural population is still below the poverty line, but the incidence of urban poverty has dropped to 10%. It is clear that urban residents have benefited more from the recent economic boom than rural (60).

## Conclusions

According to the research of the World Bank Group and WHO, there is still approximately 400 million people without access to basic health services in 2015, and 6% of the population in low- and middle-income countries fell into extreme poverty or further aggravated poverty due to medical expenditure. Based on the data of Sub-Saharan Africa from 2000 to 2019, this article uses ARDL method to explore whether the health human capital formed under the health care expenditure can effectively help the region out of the poverty trap in the long and short term.

In the long run, there is no evidence that health human capital can help the Sahara out of the poverty trap, which is closely related to local income inequality, government corruption, and inefficiency. As Roseman (1972) emphasized, a person buys medical services to maintain “good health.” Therefore, if “health” is a normal commodity, then health investment will continue to increase in the process of economic growth and may squeeze out physical capital investment, thus damaging economic growth in the long run. In the short run, the lag of poverty has a considerable stickiness to the current poverty, which reflects the self-continuity of poverty trap, and increases the difficulty of poverty reduction. Health human capital has a significant effect on getting rid of poverty. The government's medical and health expenditure is used to treat and prevent diseases, improve the living conditions of the poor, or save the high amount of medical expenditure in the household savings, which are of great help to get rid of the poverty trap. There also has a threshold effect in the poverty reduction model of healthy human capital. When the economic development level reaches the threshold, the effect of health human capital in helping Sub-Saharan areas get rid of poverty trap is more obvious and deeper. The extended Solow economic growth model also proved the views. No matter in the long- or short-term conditions, economic growth has a significant help to get rid of the poverty trap, which is consistent with the research conclusion of Gong et al. (34). The development of aging population hinders the effect of poverty reduction. Therefore, Sub-Saharan Africa must pay attention to the serious problem of aging. Education expenditure does not show significant effect for the time being, which may be related to the crowding-out effect of health expenditure.

One of the basic contents of universal health coverage is to achieve universal health protection and protect all people from health threats. Every year, millions of people fall into poverty because of the high cost of out-of-pocket health care (12). Our research also proves the significant effect of health human capital on poverty reduction. In order to get rid of the poverty trap in Sub-Saharan region, government needs to promote the national health insurance and strengthen the health system as a whole plan. When policy makers implement the medical security plan, it should strive to spread more benefits to low-income areas and bring benefits to the poor. At the same time, the government should also pay attention to improving work efficiency and integrity. At the same time, considering that health human capital will cross a threshold of economic development level and bring different effects on poverty, Sub-Saharan countries must also be aware of the important impact of health care expenditure on economic growth, so as to establish a long-term sustainable social security system.

## Data Availability Statement

The original contributions presented in the study are included in the article/supplementary material, further inquiries can be directed to the corresponding authors.

## Author Contributions

Q-SW: conceptualization, methodology, and software. Y-FH: data curation and writing—original draft preparation. RT: visualization and investigation. N-CM: writing—reviewing and editing. All authors contributed to the article and approved the submitted version.

## Conflict of Interest

The authors declare that the research was conducted in the absence of any commercial or financial relationships that could be construed as a potential conflict of interest.
